# Supplementary material for: Measurement error using a SeeMaLab structured light 3D scanner against a Microscribe 3D digitizer
Source: PeerJ. 2021 Aug 20;9:e11804. doi: 10.7717/peerj.11804 (PMC8381885; doi:10.7717/peerj.11804)
Supplement: Supplemental Information 1 — For a specific error source, Procrustes ANOVA was run on all unique paired datasets. We report Procrustes ANOVA residual R2 (Rsq) and repeatability (R) for all unique paired datasets, and compute means for each error source. For the comparison between scans, the Extended Scanner Dataset is used, whereas all other comparisons are based on the Device Comparison Dataset. [file peerj-09-11804-s001.pdf]

Table S1. Pairwise Procrustes ANOVA on shape. For a specific error source, Procrustes ANOVA was run on all unique paired datasets. We report Procrustes ANOVA residual R^2 (Rsq) and repeatability (R) for all unique paired datasets, and compute means for each error source. For the comparison between scans, the *Extended Scanner Dataset* is used, whereas all other comparisons are based on the *Device Comparison Dataset*.

**Error source**	**Dataset 1**	**Dataset 2**	**Rsq**	**R**
**Between-device**	msA1	scAA1	0.051	0.949
	msA1	scAA2	0.043	0.957
	msA2	scAA1	0.048	0.952
	msA2	scAA2	0.041	0.960
	msB1	scBB1	0.048	0.952
	msB1	scBB2	0.048	0.952
	msB2	scBB1	0.045	0.955
	msB2	scBB2	0.045	0.955
	**Mean**		**0.046**	**0.954**
**Between-operator**	msA1	msB1	0.054	0.945
	msA1	msB2	0.052	0.947
	msA2	msB1	0.053	0.947
	msA2	msB2	0.051	0.949
	scAA1	scBB1	0.032	0.968
	scAA1	scBB2	0.033	0.968
	scAA2	scBB1	0.039	0.962
	scAA2	scBB2	0.045	0.955
	**Mean**		**0.045**	**0.955**
**Within-operator**	msA1	msA2	0.013	0.987
	msB1	msB2	0.009	0.991
	scAA1	scAA2	0.024	0.977
	scBB1	scBB2	0.012	0.989
	**Mean**		**0.014**	**0.986**
**Between-scan**	scAA1	scBA1	0.021	0.979
	scAA1	scBA2	0.023	0.977
	scAA2	scBA1	0.029	0.972
	scAA2	scBA2	0.030	0.970
	scAB1	scBB1	0.015	0.985
	scAB1	scBB2	0.012	0.989
	scAB2	scBB1	0.015	0.986
	scAB2	scBB2	0.011	0.989
	**Mean**		**0.020**	**0.981**
