# Supplementary material for: Measurement error using a SeeMaLab structured light 3D scanner against a Microscribe 3D digitizer
Source: PeerJ. 2021 Aug 20;9:e11804. doi: 10.7717/peerj.11804 (PMC8381885; doi:10.7717/peerj.11804)
Supplement: Supplemental Information 2 — We applied the following nested hierarchical structure: Specimen >Device >Operator >Landmark replica. The R-squared values (Rsq) give an estimate of the relative contribution of each factor to the total shape variation. [file peerj-09-11804-s002.pdf]

**Table S2. Nested Procrustes ANOVA on shape for device comparison: Only specimens from the Baltic Sea population**. We applied the following nested hierarchical structure: Specimen > Device > Operator > Landmark replica. The R-squared values (Rsq) give an estimate of the relative contribution of each factor to the total shape variation.

| Variables | Df | SS | MS | Rsq | F | Z | Pr(>F) |
|---|---|---|---|---|---|---|---|
| ***Device Comparison Dataset*: Only specimens from the Baltic Sea population** | | | | | | | |
| Specimen | 16 | 0.348 | 0.022 | 0.894 | 184.509 | 20.325 | 0.001 |
| Specimen:Device | 17 | 0.014 | 0.001 | 0.035 | 6.803 | 16.463 | 0.001 |
| Specimen:Device:Operator | 34 | 0.020 | 0.001 | 0.050 | 4.887 | 16.615 | 0.001 |
| Residuals (Landmark replica) | 68 | 0.008 | 0.000 | 0.021 | | | |
| Total | 135 | 0.389 | | | | | |
