# Supplementary material for: Measurement error using a SeeMaLab structured light 3D scanner against a Microscribe 3D digitizer
Source: PeerJ. 2021 Aug 20;9:e11804. doi: 10.7717/peerj.11804 (PMC8381885; doi:10.7717/peerj.11804)
Supplement: Supplemental Information 3 — The Alternative Device Comparison Dataset is constructed from the datasets where each operator placed the landmarks on the 3D models reconstructed from the scans made by the other operator (scAB1, scAB2, scBA1, scBA2) and the Microscribe digitizer datasets (msA1, msA2, msB1, msB2). For a specific error source, Procrustes ANOVA was run on all unique paired datasets. We report Procrustes ANOVA residual R2 (Rsq) and repeatability (R) for all unique paired datasets, and compute means for each error source. [file peerj-09-11804-s003.pdf]

Table S3. Pairwise Procrustes ANOVA on shape: Alternative definition of dataset. The *Alternative Device Comparison Dataset* is constructed from the datasets where each operator placed the landmarks on the 3D models reconstructed from the scans made by the other operator (scAB1, scAB2, scBA1, scBA2) and the Microscribe digitizer datasets (msA1, msA2, msB1, msB2). For a specific error source, Procrustes ANOVA was run on all unique paired datasets. We report Procrustes ANOVA residual R^2 (Rsq) and repeatability (R) for all unique paired datasets, and compute means for each error source.

Error source	Dataset 1	Dataset 2	Rsq	R
**Between-device**	msA1	scBA1	0.055	0.944
	msA1	scBA2	0.055	0.945
	msA2	scBA1	0.054	0.946
	msA2	scBA2	0.054	0.946
	msB1	scAB1	0.045	0.955
	msB1	scAB2	0.047	0.953
	msB2	scAB1	0.043	0.957
	msB2	scAB2	0.043	0.957
	**Mean**		**0.049**	**0.950**
**Between-operator**	msA1	msB1	0.054	0.945
	msA1	msB2	0.052	0.947
	msA2	msB1	0.053	0.947
	msA2	msB2	0.051	0.949
	scBA1	scAB1	0.035	0.966
	scBA1	scAB2	0.035	0.966
	scBA2	scAB1	0.039	0.962
	scBA2	scAB2	0.038	0.963
	**Mean**		**0.044**	**0.956**
**Within-operator**	msA1	msA2	0.013	0.987
	msB1	msB2	0.009	0.991
	scBA1	scBA2	0.018	0.982
	scAB1	scAB2	0.008	0.992
	**Mean**		**0.012**	**0.988**
