# Supplementary material for: Measurement error using a SeeMaLab structured light 3D scanner against a Microscribe 3D digitizer
Source: PeerJ. 2021 Aug 20;9:e11804. doi: 10.7717/peerj.11804 (PMC8381885; doi:10.7717/peerj.11804)
Supplement: Supplemental Information 4 — The Alternative Device Comparison Dataset is constructed from the datasets where each operator placed the landmarks on the 3D models reconstructed from the scans made by the other operator (scAB1, scAB2, scBA1, scBA2) and the Microscribe digitizer datasets (msA1, msA2, msB1, msB2). We applied the following nested hierarchical structure: Specimen >Device >Operator >Landmark replica. (A) All datasets. (B) Only scanner-based datasets. (C) Only digitizer-based datasets. The R-squared values (Rsq) give an estimate of the relative contribution of each factor to the total shape variation. Repeatabilities are for landmark replica only. [file peerj-09-11804-s004.pdf]

Table S4. Nested Procrustes ANOVA on shape for device comparison: Alternative definition of dataset. The *Alternative Device Comparison Dataset* is constructed from the datasets where each operator placed the landmarks on the 3D models reconstructed from the scans made by the other operator (scAB1, scAB2, scBA1, scBA2) and the Microscribe digitizer datasets (msA1, msA2, msB1, msB2). We applied the following nested hierarchical structure: Specimen > Device > Operator > Landmark replica. **(A)** All datasets. **(B)** Only scanner-based datasets. **(C)** Only digitizer-based datasets. The R-squared values (Rsq) give an estimate of the relative contribution of each factor to the total shape variation. Repeatabilities are for landmark replica only.

Variables	Df	SS	MS	Rsq	F	Z	Pr(>F)	R
**A.** ***Alternative Device Comparison Dataset***								
Specimen	21	0.616	0.029	0.923	373.847	21.408	0.001	
Specimen:Device	22	0.018	0.001	0.027	9.133	20.399	0.001	
Specimen:Device:Operator	44	0.025	0.001	0.038	6.387	20.723	0.001	
Residuals (Landmark replica)	88	0.008	0.000	0.012				
Total	175	0.668						
**B.** ***Alternative Device Comparison Dataset*****: Scanner**								
Specimen	21	0.314	0.015	0.957	154.398	18.171	0.001	0.987
Specimen:Operator	22	0.010	0.000	0.030	4.602	11.851	0.001	
Residuals (Landmark replica)	44	0.004	0.000	0.013				
Total	87	0.329						
**C.** ***Alternative Device Comparison Dataset*****: Digitizer**								
Specimen	21	0.315	0.015	0.942	177.721	19.191	0.001	0.989
Specimen:Operator	22	0.016	0.001	0.047	8.441	25.747	0.001	
Residuals (Landmark replica)	44	0.004	0.000	0.011				
Total	87	0.334						
