# Supplementary figures and images for: Measurement error using a SeeMaLab structured light 3D scanner against a Microscribe 3D digitizer
Source: PeerJ. 2021 Aug 20;9:e11804. doi: 10.7717/peerj.11804 (PMC8381885; doi:10.7717/peerj.11804)

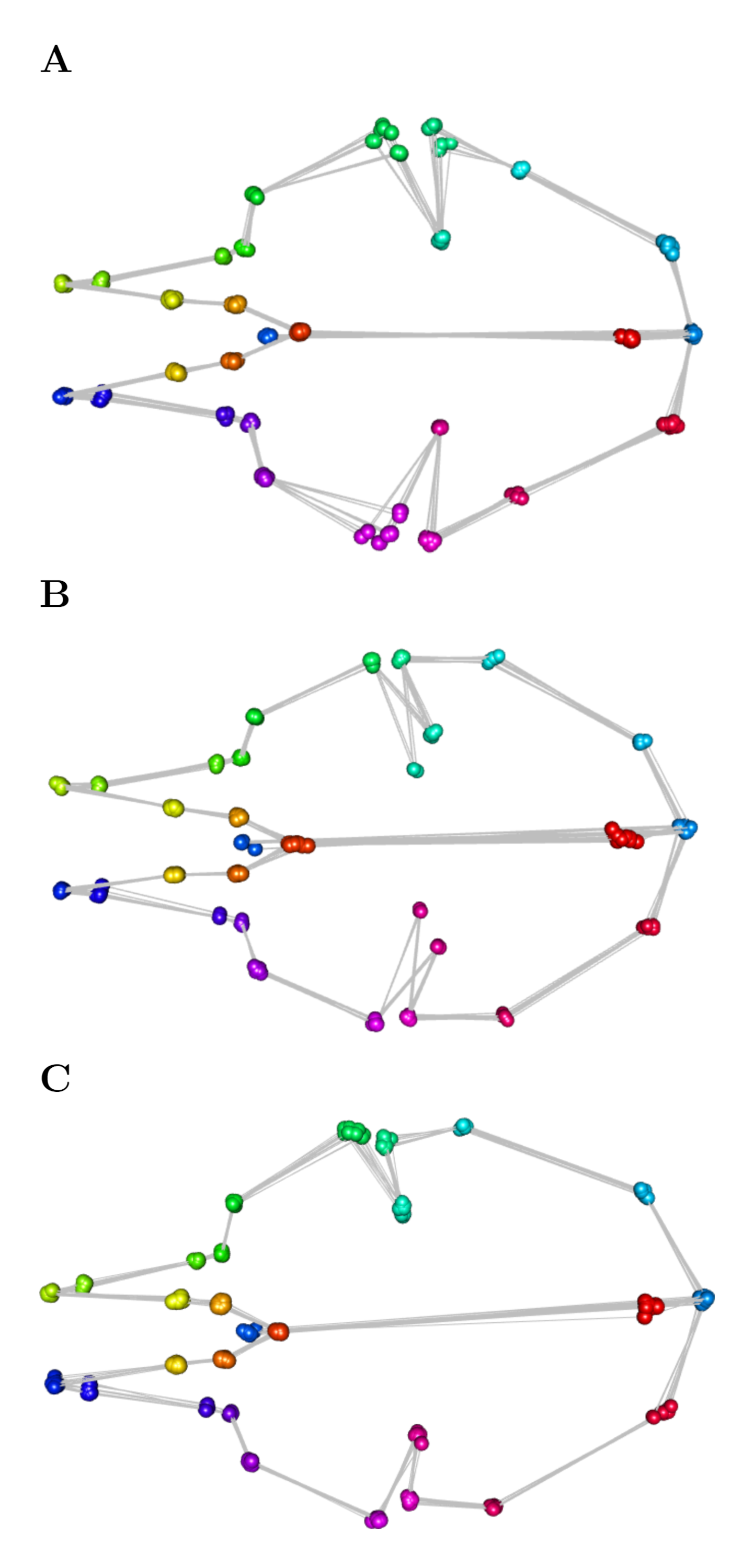

Supplement: Supplemental Information 5 — Computation of Procrustes distances comparing methods, between-operator, scan replica, and within-operator, and subsequent analysis of outliers showed that landmark annotation of the three skulls 96, 42.23 and 14 contributed to 87.5% of the observed outliers in the analysis of Procrustes distances. The figures show all landmark configurations for these three skulls (combined Device Comparison Dataset and Extended Scanner Dataset). Only a few landmark coordinates exhibit large variation. (A) Skull 96, which contributed to 40.6% of the observed outliers. The variation is largest for landmarks 12/26 and 13. This is the second-largest skull in the sample. (B) Skull 42.23, which contributed to 34.4% of the observed outliers. The variation is largest for landmarks 1 and 14/28. This is a skull of medium-size. (C) Skull 14, which contributed to 12.5% of the observed outliers. The variation is largest for landmarks 12, 14/28 and 31. This is the smallest skull in the sample. Interactive WebGL figures are available online at http://eco3d.compute.dtu.dk/seals. [file peerj-09-11804-s005.png]

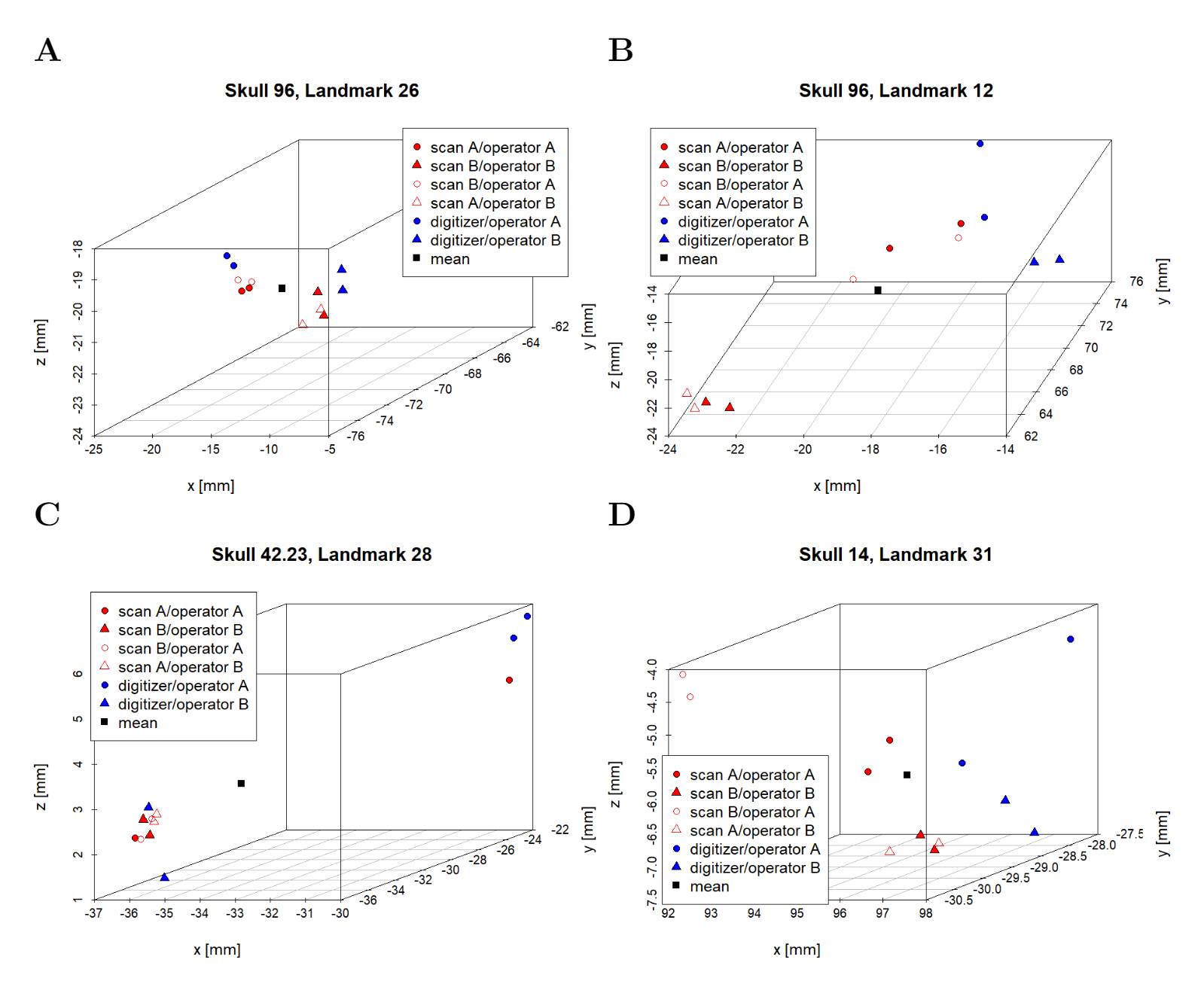

Supplement: Supplemental Information 6 — Computation of Procrustes distances comparing methods, between-operator, scan replica, and within-operator, and subsequent analysis of outliers showed that landmark annotation the three skulls 96, 42.23 and 14 contributed to 87.5% of the observed outliers in the analysis of Procrustes distances. Only a few landmark coordinates exhibit large variation. The figures show landmark coordinates for some examples of landmarks with a large variation on one of these skulls (combined Device Comparison Dataset and Extended Scanner Dataset). We observe differences between operators and devices, or a combination of them. We used unscaled GPA. Aberrations range from a few millimetres to about 1.5 centimetres (for a given direction). (A) Landmark 26 on skull 96. There are large differences between operators and smaller differences between devices. The jugaltemporal suture was fully fused on this skull. (B) Landmark 12 on skull 96. There is a large spread between devices for operator B, and in general differences between operators, and between devices. The jugaltemporal suture was fully fused on this skull. (C) Landmark 28 on skull 42.23. There is a large spread between landmarks placed by operator A. This landmark was noted to be difficult to place. (D) Landmark 31 on skull 14. There is a difference both between device and operators, and between scans for operator A. It was noted that there was no clear apex. [file peerj-09-11804-s006.png]
