# Supplementary material for: Measurement error using a SeeMaLab structured light 3D scanner against a Microscribe 3D digitizer
Source: PeerJ. 2021 Aug 20;9:e11804. doi: 10.7717/peerj.11804 (PMC8381885; doi:10.7717/peerj.11804)
Supplement: Supplemental Information 7 [file peerj-09-11804-s007.pdf]

Data Collection Protocol

Dolores Messer[1], Michelle Strecker Svendsen[2], Anders Galatius[3], Morten Tange Olsen[2], Vedrana Andersen Dahl[1], Knut Conradsen[1], and Anders Bjorholm Dahl[1]

[1]**Technical University of Denmark, Department of Applied Mathematics and Computer Science, Kongens Lyngby, Denmark**
[2]**University of Copenhagen, Globe Institute, Copenhagen, Denmark**
[3]**Aarhus University, Department of Bioscience, Roskilde, Denmark**

Corresponding author:
Dolores Messer[1]

Email address: dolmes@dtu.dk

INTRODUCTION

The purpose of our study was to compare the performance of a 3D structured light scanning setup (SeeMaLab (Eiriksson et al., 2016)) with a Microscribe digitizer by capturing the shape variation of 22 grey seal skulls, which is measured at 31 fixed anatomical landmarks. For a given skull, Cartesian coordinates of the landmarks were recorded by means of two different measurement methods: Direct measurement on the physical skull using a digitizer, and placement of the landmarks on the 3D digital model, which was reconstructed from surface scans of the skull. This document describes in detail how the data was acquired, allowing replication of our measurements. We further discuss the limitations and possible improvements of the scanner pipeline, as we intend to use the obtained high-quality 3D models for future shape analysis.

3D DIGITIZER

The 3D digitizer used was a Microscribe® 3D digitizer (Immersion Corporation, San Jose, CA, USA; https://www.immersion.com). Landmark coordinates were automatically imported in a Microsoft Excel spreadsheet when pressing a foot pedal by using the Microscribe Utility software version 5.0.0.1. The measurement setup is shown in Fig. 1. Between repetitions of landmark measurements, the position of the skull relative to the digitizer remained unchanged. Moreover, a few minutes breaks were inserted between repeated measurements in order to make them as independent as possible. It took between 5-10 minutes to collect a full set of 31 fixed landmark coordinates on a skull.

3D STRUCTURED LIGHT SCANNER PIPELINE

The pipeline consisted of three steps: First, a specimen was scanned with a 3D structured light scanner, resulting in a series of point clouds. Afterwards, a triangle mesh of the surface was reconstructed from the point clouds, yielding a 3D digital model of the specimen. Finally, landmark coordinates were measured on the 3D model.

Scanning

We used a 3D structured light scanning setup (SeeMaLab). The SeeMaLab scanner is build by a research group at the Technical University of Denmark as described in Eiriksson et al. (2016). The portable scanner consists of a Dell Precision M 4800 laptop, two PtGray Grashopper 9.1MP cameras, a LG projector, a mounting rig and camera tripod, a motor-driven rotation stage that allows for a 360° acquisition, and a calibration plate. Figure 2 shows the typical setup of the SeeMaLab scanner. Accuracy of the SeeMaLab scanner was evaluated according to VDI 2634 (The Association of German Engineers (VDI), 2012) and is below 150 μm. The absolute accuracy and precision of the SeeMaLab scanner is comparable to high-end commercial metrology grade scanners[1] (Eiriksson et al., 2016). The scanner uses open-source, giving full insight into the post-processing of the acquired data, which is difficult when using a commercial scanner.

[1]GOM ATOS III Triple Scan, https://www.capture3d.com/3d-metrology-solutions/3d-scanners/atos-triple-scan

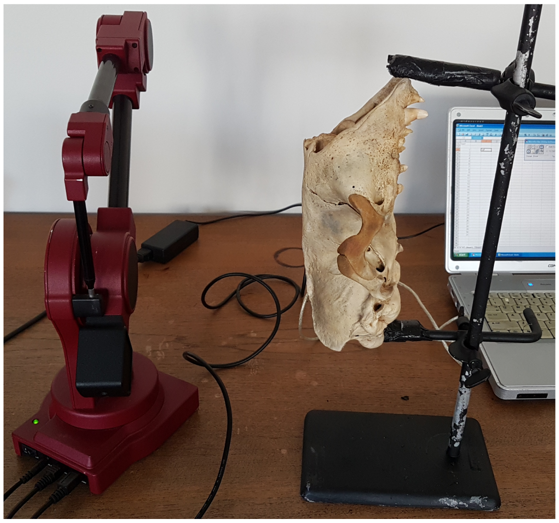

Figure 1. Measurement using a 3D digitizer. A skull is positioned vertically on a stand in order to be able to reach all landmarks without one having to move the skull. The digitizer is placed on the dorsal side of the skull. The setup is the same for all skulls.

Trinderup et al. (2016) found a sub-millimetre difference when comparing a structured light scan obtained with the SeeMaLab scanner with a μCT scan of a fox and bulldog skull.

The SeeMaLab scanner includes a rotation stage which allows for automatically rotating the skull and scanning from a set of predefined directions covering the full 360°. For each scan direction, a series of images are collected to create a dense set of 3D points. Combining the point clouds from these sub-scans results in a set of 3D points that is fully covering the skull in the given position. Image acquisition consists of projecting a sequence of patterns onto the object to scan. Obtained images are then converted to depth values by means of the specific algorithm. For our purpose of scanning grey seal skulls, we chose the phase shifting pattern mode.

Calibration was done using a checkerboard plate (size: 22×13, cell: 15 mm). Calibration parameters consist of camera focal lengths, central points, lens distortion parameters, camera extrinsics (their relative position and angles), and the location and orientation of the rotation stage axis. The system was calibrated at least once per day.

To reduce background noise, all the holders (stand, cork holders and rigid foam) were sprayed with matt black colour, and the wall behind the rotation stage, the rotational stage itself, and the table below the rotation plate were covered with matt black cloth.

The goal of scanning grey seal skulls was to obtain 3D models that accurately capture the full shape of the skull. This was measured from 31 fixed landmarks. Since the surface scan obtained from one rotation only covered parts of the skull, it was necessary to place the skull in differnet positions and make a scan from each position, which then were combined to make up the 3D surface model. To ensure optimal coverage of the skull surface, a pre-study was conducted to identify the different scan positions, and their respective angular span and steps. The pre-study showed that four positions were needed: one dorsal view with the skull resting on the ventral side (Fig. 3A), one view around the anteroposterior axis obtained by mounting the skull vertically in a stand for stability reasons (Fig. 3B), and two ventral views with the skull tipped to the side, resting on landmarks 13, and 27, respectively (Fig. 3C and 3D), where small cork holders were used to increase stability. The typical scanning time of a specimen was about 20 minutes for all four positions.

In order to avoid recalibration between scanning of different positions or specimens, the scanner was placed so that a skull was inside the field of view of both cameras for all positions, similarly-sized specimens were scanned after each other, and the amount of light was exclusively controlled by the camera exposure time (camera aperture was set at the smallest possible value). When scanning a new specimen, the operator thus adjusted camera exposure time and the position of the cameras with respect to the specimen to control for differences in colour, brightness and size between the individual specimens, and obtain a scan of good quality. Typically, exposure time was changed for each specimen, whereas

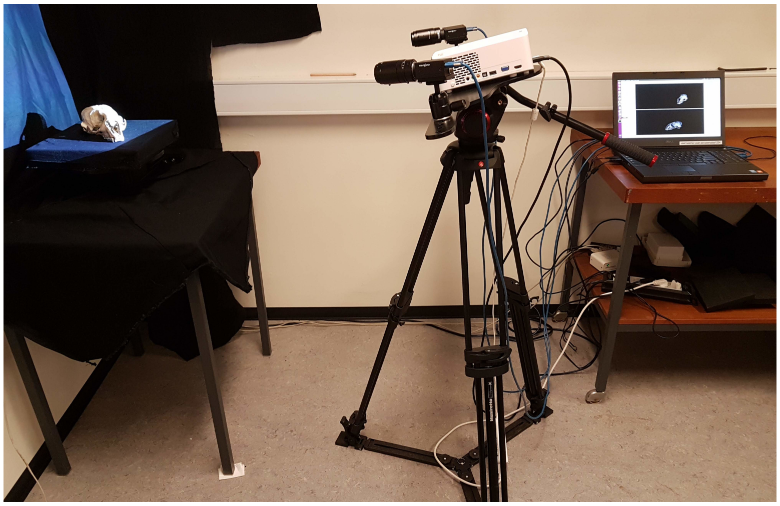

Figure 2. SeeMaLab scanner setup. Scanning a grey seal skull at the Natural History Museum in Copenhagen.

the camera position was only adjusted for larger size differences. Exposure time determines the amount of light that reaches the image, and is a parameter that needed careful tuning to scan darker parts of a skull properly, while not overexposing brighter parts. In general, especially scanning of the teeth was challenging, as they were typically much brighter than the rest of the skull. However, none of the considered landmarks were located on the teeth, only a few were on the border between a tooth and bone. Thus, to be able to scan the regions of interest, the teeth were sometimes overexposed. Another factor, which affected the scanning quality of the teeth, was that a few teeth were loose.

3D model reconstruction

After having scanned a specimen in four different positions, a 3D model was generated. Before alignment, we improved the quality of the 3D point sets by pre-processing. To avoid misalignment between scanning positions on skulls with loose teeth, the point measurements on loose teeth were removed manually from the point clouds from three positions using the open-source system MeshLab version 2016 (Italian National Research Council, Pisa, Italy; https://www.meshlab.net/; Cignoni et al., 2008). We further observed that the teeth sometimes were out of focus when scanning, which was related to scanning bright (overexposed) teeth against a dark background. This resulted in poorer localisation of the teeth surface and was expressed in point clouds with wider teeth. Most often, scans of a skull placed horizontally (Fig. 3A) were affected, a few times also scans of a skull placed on landmark 13 or 27 (Fig. 3C and 3D). In such a case, to avoid misalignment, the teeth were removed manually from the resulting point cloud of the affected position using MeshLab.

Alignment of the four point clouds corresponding to the different scanned positions of a given specimen was done in several steps. First, a global alignment was obtained by pairwise aligning the point clouds, where each pairwise alignment involved global RANSAC registration based on FPFH feature matching (Rusu et al., 2009), followed by ICP registration with colour aware energy term (Park et al., 2017) for refining the alignment. For this first step, we used the Open3D library (Zhou et al., 2018). The global rigid registration was then used to initialize the non-rigid alignment of all partial point clouds as suggested by Gawrilowicz and Bærentzen (2019). The resulting point cloud was manually cleaned around the teeth in MeshLab to coarsely remove points resulting from reflection. The final 3D model was reconstructed by means of Poisson surface reconstruction (Kazhdan et al., 2006; Kazhdan and Hoppe, 2013) using the Adaptive Multigrid Solvers software version 12.00 by Kazhdan (Johns Hopkins University, Baltimore, MA, USA; http://www.cs.jhu.edu/~misha/Code/PoissonRecon/Version12.00).

The fully automated registration procedure was running for one to two hours per specimen, depending on skull size. A few specimen required no pre-processing, whereas the remaining skulls required a time-intensive pre-processing of the teeth, which typically took about half an hour for one specimen. Cleaning of the final point cloud could typically take up to an hour for one specimen.

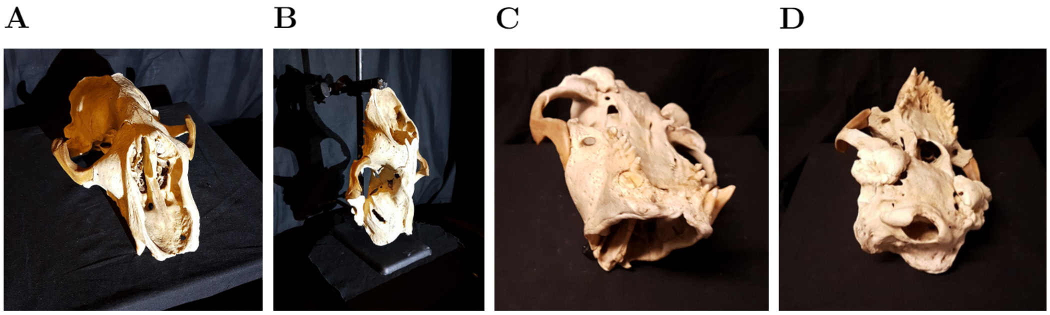

Figure 3. **Scanning positions of grey seal skulls.** Every skull was scanned in four different positions: **(A)** Skull placed horizontally (360° scan in steps of 30°). This position provided detailed acquisition of the lateral sides, the caudal structures, nasal and maxilla bones, and the external part of the jugal/zygomatic bones. **(B)** Skull mounted vertically on stand (360° scan in steps of 20°). This position allowed a detailed acquisition of the dorsal side, the ventral side, part of the lateral sides, and the internal part of the jugal/zygomatic bones. **(C)** Skull placed on landmark 13 (180° scan in steps of 30°). **(D)** Skull placed on landmark 27 (180° scan in steps of 30°). The positions shown in **(C)** and **(D)** provided details of the ventral side and the back. It has to be stressed that a scan of the position shown in **(B)** was not fully covering the ventral side due to projected shades.

Measurement of landmark coordinates

The landmarks were annotated by the operators on the reconstructed 3D digital models using the Stratovan Checkpoint software version 2018.08.07 (Stratovan Corporation, Davis, CA, USA; https://www.stratovan.com/products/checkpoint). Landmark annotation of one 3D model took about 10-15 minutes.

Discussion

As mentioned earlier, using an open-source system had the advantage of having full control over the scanning procedure. Thus, using the SeeMaLab scanner allowed us to decide ourselves how to clean the noisy raw scans, and which reconstruction algorithm we wanted to use. However, in contrast to commercial scanners, the SeeMaLab scanner has mainly been used as a research platform so far, thus, it came with some practical issues that would have to be improved to make it ready for large-scale use.

One of the limitations was that an operator needed to have comprehensive knowledge of how to acquire a scan and how to reconstruct 3D models from the raw scans. However, this knowledge could be gained by a few days of training. Reconstructing the 3D models based on the scanner output included several manually controlled steps using algorithms and software of an operator's choice. These steps could in principle be automated such that the operator can choose from a selection of reconstruction algorithms.

As mentioned before, another issue we observed were reflection and focus problems at the teeth, which was reflected in outliers in the point cloud, and slightly wider teeth. Automated quality control of the scanned points could be implemented to detect and remove outliers, instead of leaving this to the operator, as this step was quite time-consuming and required some knowledge. In order to avoid overexposition, the use of multiple exposure times for scanning a skull, which is an example of high-dynamic range techniques, could be implemented.

It has to be noted that the colour model of the resulting 3D digital models was imperfect. Even though this did not affect geometry, it might have affected placement of landmarks that are actually located at points with a large colour gradient (e.g. on the border between teeth and bone), as this colour gradient might not have been seen on the 3D models due to the imperfect colour model. However, the colour model could be improved.

In general, the process of scanning a skull and reconstructing the final 3D models was quite time-intensive (a few hours in total, 20 minutes for scanning). This process could be substantially improved by automating the pipeline.

It has to be stressed that if different operators scan the same skull following the above described scanning protocol, their scans differ for several reasons. There are e.g. variations in calibration or illuminations, and possibly also differences due to the operators respective choice of the parameters exposure time and camera position with respect to the object to be scanned. However, the differences in captured geometry are expected to be minute. Exposure time determines the amount of light that reaches the image, and overexposition can lead to reflection and out of focus problems, whereas underexposed regions are not scanned. The distance between the object to scan and the cameras determines the density of acquired point sets. If the distance between the cameras and the specimen is large, details might not be seen. On the other hand, if the scanned object is placed such that it fills the field of view of the cameras, measurement error due to optical distortion is larger. Thus, the two parameters exposure time and distance between the cameras and object to scan have to be determined carefully.

While the purpose of our study was to compare the SeeMaLab scanner with a Microscibe digitizer, we also wished to produce some high-quality 3D models for future shape analysis, and to establish possible improvements of the scanner. To produce high-quality models, we probably used more time on pre-processing and cleaning than needed if we only had been interested in annotating 31 landmarks on the 3D models. To prioritize between possible improvements, we thoroughly investigated the imperfections of a 3D surface scan. But already now, the SeeMaLab scanner is capable of producing 3D surface models which can be used for geometric morhpmetrics.

REFERENCES

Cignoni, P., Callieri, M., Corsini, M., Dellepiane, M., Ganovelli, F., and Ranzuglia, G. (2008). MeshLab: an Open-Source Mesh Processing Tool. In Scarano, V., Chiara, R. D., and Erra, U., editors, *Eurographics Italian Chapter Conference*. The Eurographics Association.

Eiriksson, E., Wilm, J., Pedersen, D., and Aanæs, H. (2016). Precision and accuracy parameters in structured light 3-D scanning. *International Archives of the Photogrammetry, Remote Sensing and Spatial Information Sciences*, XL-5/W8:7–15.

Gawrilowicz, F. and Bærentzen, J. (2019). Optimal, non-rigid alignment for feature-preserving mesh denoising. In *Proceedings of the International Conference on 3D Vision*, United States. IEEE. 2019 International Conference on 3D Vision, 3DV 2019; Conference date: 16-09-2019 Through 19-09-2019.

Kazhdan, M., Bolitho, M., and Hoppe, H. (2006). Poisson surface reconstruction. In *Eurographics Symposium on Geometry Processing*, SGP '06, page 61–70, Goslar, DEU. Eurographics Association.

Kazhdan, M. and Hoppe, H. (2013). Screened poisson surface reconstruction. *ACM Transactions on Graphics*, 32(3).

Park, J., Zhou, Q., and Koltun, V. (2017). Colored point cloud registration revisited. In *2017 IEEE International Conference on Computer Vision (ICCV)*, pages 143–152.

Rusu, R. B., Blodow, N., and Beetz, M. (2009). Fast Point Feature Histograms (FPFH) for 3D registration. In *2009 IEEE International Conference on Robotics and Automation*, pages 3212–3217.

The Association of German Engineers (VDI) (2012). *VDI-Standard: VDI/VDE 2634 Part 2, Optical 3-D measuring systems - Optical systems based on area scanning*. Beuth Verlag, Berlin.

Trinderup, C. H., Dahl, V. A., Gregersen, K. M., Orlando, L. A. A., and Dahl, A. B. (2016). The traveling optical scanner – case study on 3D shape models of ancient Brazilian skulls. In Mansouri, A., Nouboud, F., Chalifour, A., Mammass, D., Meunier, J., and Elmoataz, A., editors, *Image and Signal Processing*, pages 398–405, Cham. Springer International Publishing.

Zhou, Q.-Y., Park, J., and Koltun, V. (2018). Open3D: A modern library for 3D data processing.
